# Riedel's Lobe of the Liver: A Case Report

**DOI:** 10.1097/MD.0000000000000430

**Published:** 2015-01-26

**Authors:** Christos Savopoulos, Nikolaos Kakaletsis, Georgia Kaiafa, Fotios Iliadis, Anna Kalogera-Fountzila, Apostolos I. Hatzitolios

**Affiliations:** From the First Propedeutic Department of Internal Medicine (CS, NK, GK, FI, AIH), Medical School; and Department of Radiology (AK-F), Medical School, Aristotle University of Thessaloniki, AHEPA University Hospital, Thessaloniki, Greece.

## Abstract

Riedel lobe of the liver is a simple anatomical variation, a downward tongue-like projection of the anterior edge of the right lobe of the liver to the right of the gallbladder with its typical case to be rare.

We report the case of a 71-year-old woman with typical feature of a nonpalpable Riedel's lobe of the liver, as an incidental finding who was referred for reported hypergammaglobulinemia (22.7% [9%–19%]). Both features were attributed to a chronic inflammation because of an abscess in the right iliopsoas caused by infection due to bilateral hip replacement which underwent revision surgery. This was confirmed by her medical history, the imaging findings combined with elevated C-reactive protein, and by cross-reaction weak positive autoantibodies.

Generally, knowledge or suspicion of Riedel's lobe of the liver is important, as it does not always remain clinically latent, as in our case, and it can be complicated by its torsion or hepatic tumors.

## INTRODUCTION

The liver is the largest internal organ in the human body. Its size, either in clinical examination or in imaging techniques, depends on several factors such as age, sex, body size, and shape, and the particular examination technique utilized. In some cases, the liver can be palpable due to anatomic reasons or underlying abnormal conditions.^1^ In clinical practice, congenital abnormalities of the liver are comparatively rare.^2^

Riedel lobe of the liver is a simple anatomical variation, a downward tongue-like projection of the anterior edge of the right lobe of the liver to the right of the gallbladder.^3^ It was originally reported by Riedel in 7 female patients who had palpable masses in the right hypochondrium, which were subsequently confirmed at surgery.^4^ The clinical significance of Riedel lobe has been identified, as it is included in the differential diagnosis of right-sided abdominal palpable masses.^5^

The reported incidence of Riedel’ lobe in the general population considerably varies (3.3%–31%), which could be attributed to the uncertain criteria and multiple diagnostic methods, although the typical case is rare.^5–7^

We report the case of a 71-year-old woman with typical feature of a nonpalpable Riedel lobe of the liver, as incidental finding.

## CASE REPORT

A 71-year-old woman was referred for reported hypergammaglobulinemia (22.7% [9%–19%]). Medical history included bilateral primary and revision (due to infection) hip replacement, with the last procedure 4 years ago. Physical examination was unremarkable and the patient was afebrile without any palpated mass in her abdomen.

On admission, laboratory findings were as follows: white blood cell count 6.03 × 10^3^ cells/μL (3.8–10.5 cells/μL) (neutrophils 54% [45%–75%], lymphocytes 31.5% [20%–51%], monocytes 9% [2%–11%], eosinophils 3.5% [0.5%–10%], basophils 1.3% [0%–2%]), red blood cell count 4.44 × 10^3^ cells/μL (3.8–5.3 cells/μL), hematocrit value 38.3% (37%–47%), hemoglobin value 11.9 g/dL (12–16 g/dL), mean corpuscular volume 86.3 fL (80–99 fL), mean corpuscular hemoglobin concentration 31.1 g/dL (32–35 g/dL), reticulocytes 1.18% (0.2%–2%), platelet count 456 × 10^3^ cells/μL (150–450 cells/μL), aspartate transaminase 11 U/L (<38 U/L), alanine transaminase 6 U/L (<40 U/L), gamma-glutamyl transferase 8 U/L (5–36 U/L), alkaline phosphatase 77 U/L (35–104 U/L), creatine phosphokinase 50 U/L (0–167 U/L), lactate dehydrogenase 311 U/L (240–480 U/L), bilirubin 0.41 mg/dL (0–1 mg/dL), total proteins 7.4 g/dL (6.6–8.7 g/dL), serum albumin 3.8 g/dL (3.5–4.8 g/dL), globulins 3.6 g/dL (2.2–4 g/dL), iron (Fe) 38 μg/dL (37–158 μg/dL), ferritin 164.1 ng/mL (13–150 ng/mL), complement component 3 137 mg/dL (79–152 mg/dL), complement component 4 25.4 mg/dL (16–38 mg/dL), erythrocyte sedimentation rate 46 mm (0–20 mm), procalcitonin 0.5 ng/mL (<0.5 ng/mL), and C-reactive protein (CRP) 3.45 mg/dL (0–0.8 mg/dL). There were found positive antinuclear antibodies, weak-positive anti-DNA and anti-smooth muscle antibodies. All components in serum protein electrophoresis were slightly elevated and there was not detected abnormal protein fraction in serum and urine immunoelectrophoresis. Tumor markers and other serological tests were negative.

**Figure 1:**
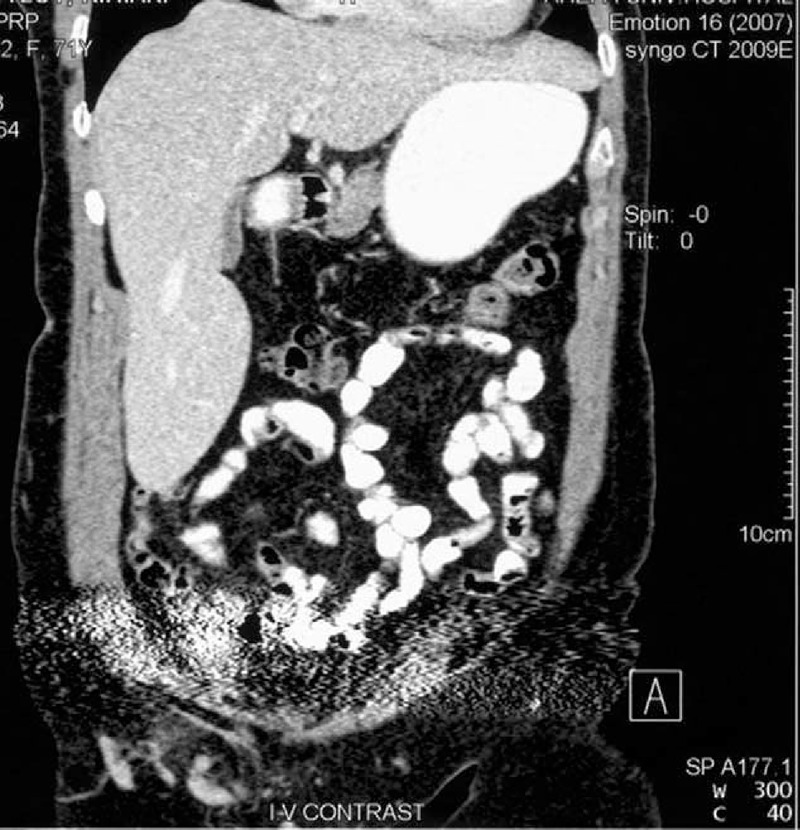
Riedel lobe as homogeneous mass (24 cm) pedunculated from the right lobe of the liver.

**Figure 2:**
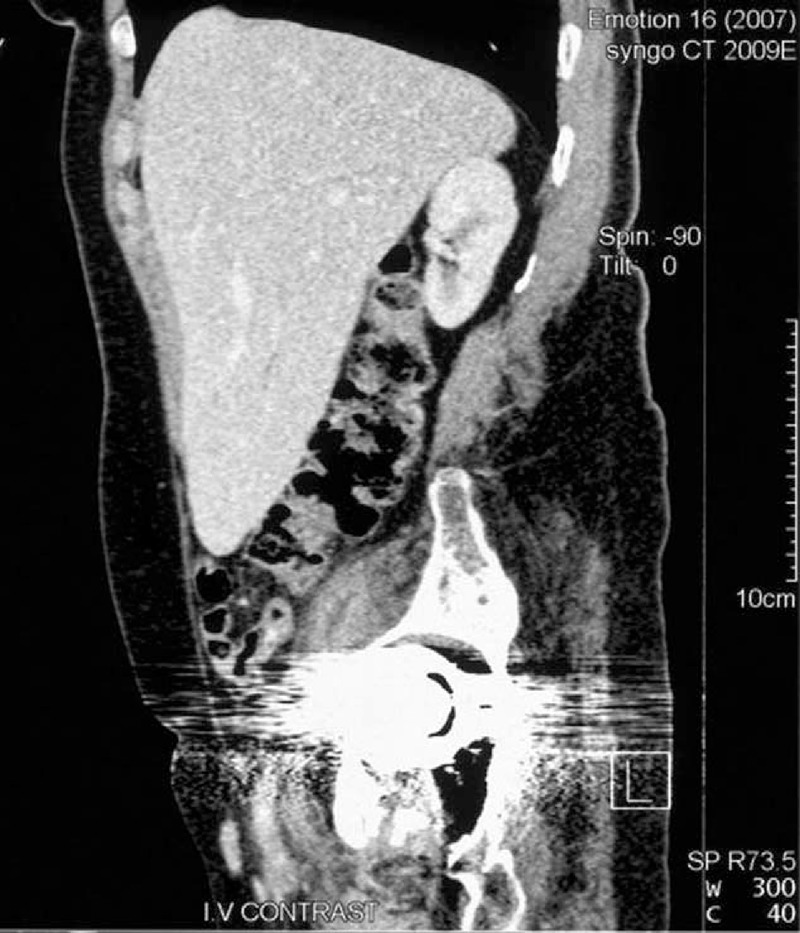
Downward elongation of the liver, ending inferior to the iliac crest.

Thoracoabdominal computed tomography (CT) scan in addition to many artifacts due to bilateral hip replacement revealed a collection in the right iliopsoas (abscess or hematoma) and a homogeneous mass (24 cm) pedunculated from right lobe of the liver, having the same density, which was elongated downward, ending inferior to the iliac crest (Figures 1 and 2). Both features were depicted having the same size in a CT scan, 3 years ago.

A diagnosis of Riedel lobe of the liver was made. She was discharged from our hospital without treatment for the abdominal mass with recommendation for reassessment in 3 months.

## DISCUSSION

This rare morphologic feature of hepatic lobulation was firstly described by Corbin in 1830 and it was defined by Riedel in 1888, as a “round tumor on the anterior side of the liver, near the gallbladder, to its right.” In the literature, it is also referred as floating lobe, “tongue like,” or constriction lobe.^4^ Nowadays, this downward elongation of the liver is frequently observed (mostly in women) by modern imaging techniques, but the typical case of Riedel lobe is rare.^4,7^

The etiology of Riedel lobe has been proposed to be either congenital or acquired. The congenital origin of Riedel lobe is supported by a congenital disembrioplasic anomaly in the development of a hepatic bud, which can lead to the formation of accessory lobes, in infrahepatic positions.^2,7^ However, Riedel attributed its appearance to the tractions exercised by the adherential syndrome due to lithiasic cholecystitis.^4^ Additionally, it is proposed that it could be in the framework of hepatic modifications caused by age or by skeletal anomalies such as ciphoscoliosis with wide thorax^7,8^ or secondary to intraperitoneal or intrapelvic inflammation or to surgical interventions.^9^

Our above reported case was a typical feature of a nonpalpable Riedel lobe of the liver, as incidental finding during the examination for hypergammaglobulinemia. Both features were attributed to a chronic inflammation because of an abscess in the right iliopsoas caused by infection due to bilateral hip replacement which underwent revision surgery. This could be explained by the medical history, the imaging findings combined with elevated CRP, and by cross-reaction weak-positive autoantibodies.

Generally, Riedel lobe can be presented with minor symptoms such as an abdominal discomfort due to the extrinsic compression and torsion episodes or without any, as in our case. Its differential diagnosis includes all causes of palpable normal liver such as emphysema, right-sided pleural effusion, congestive heart failure, thin body carriage, and deep diaphragmatic excursion or other liver diseases such as cirrhosis, hepatic, or metastatic cancer.

For its diagnosis can be used all available imaging techniques such as ultrasound (US), CT, magnetic resonance imaging, and in some cases radionuclide imaging and arteriographic examinations.^10^ Hepatic US is useful in discovering the lesion, which combined with the Doppler examination can depict its vascular or cystic features.

Typical Riedel lobe usually has good prognosis considering the early-stage diagnosis, the lack of complications, and the proper treatment such as the resection of the hypertrophic parenchyma in case of torsion with noisy clinical presentation, metastatic lesion, or hepatic hydatide cysts of the Riedel lobe.^7,11,12^ An additional management of this normal anatomical feature is proposed to consider it as a possible source of a “living-related” hepatic transplant.

Knowledge or suspicion of its possibility is important, as it does not always remain clinically latent in case of its torsion or hepatic tumors including metastasis or hepatocellular carcinoma may sometimes arise only in the lowest part of Riedel lobe.^11,13^
